# Behavior and Mechanisms of Antimony Precipitation from Wastewater by Sulfate-Reducing Bacteria *Desulfovibrio desulfuricans*

**DOI:** 10.3390/toxics13010017

**Published:** 2024-12-26

**Authors:** Fan Zhuang, Xiaowu Xiang, Jin Hu, Jing Xiong, Teng Zhang, Lei Zhou, Guoping Jiang, Min Zhang, Zhenghua Liu, Huaqun Yin, Ling Xia, Ibrahim Ahmed Ibrahim Mahmoud, Delong Meng

**Affiliations:** 1 Key Laboratory of Biometallurgy, Ministry of Education, School of Minerals Processing and Bioengineering, Central South University, Changsha 410083, China; fanzhuang@csu.edu.cn (F.Z.); 32210242@csu.edu.cn (J.H.); 19310085004@163.com (J.X.); zt18974215888@163.com (T.Z.); 205606002@csu.edu.cn (M.Z.); liuzhenghua2017csu@163.com (Z.L.); yinhuaqun_cs@sina.com (H.Y.); 2 Dongkou County Agricultural Bureau, Shaoyang 422300, China; m13278883049@163.com; 3 Hunan Urban and Rural Environmental Construction Co., Ltd., Changsha 410118, China; 4 Beijing Research Institute of Chemical Engineering and Metallurgy, Beijing 101148, China; lzhou0105@126.com (L.Z.); jiangguoping1984@163.com (G.J.); 5 Hubei Key Laboratory of Mineral Resources Processing and Environment, School of Resources and Environmental Engineering, Wuhan University of Technology, Luoshi Road 122, Wuhan 430070, China; xialing@whut.edu.cn; 6 Central Metallurgical Research and Development Institute, Cairo 11421, Egypt; ibrahimcmrdi@yahoo.ca

**Keywords:** antimony pollution, sulfate-reducing bacteria, Sb, XPS, SEM-EDX

## Abstract

The development of the non-ferrous metal industry is generating increasingly large quantities of wastewater containing heavy metals (e.g., Sb). The precipitation of heavy metals by microorganisms involves complex mechanisms that require further investigation to optimize bioremediation technologies. In this study, we employed a sulfate-reducing bacteria (SRB) strain *Desulfovibrio desulfuricans* CSU_dl to treat the antimony (Sb)-containing wastewater; the behavior of Sb and mechanisms underlying precipitation were investigated by characterizing the precipitates. The results showed that the abiotic factors constraining SRB bacterial growth greatly affect Sb forms and precipitation. For instance, Sb precipitation maximumly occurred at pH 6 and 7, or C:N ratio of 10:1 and 40:3 for Sb(III) and Sb(V), respectively, resulting in a maximum Sb removal rate of 94%. Interestingly, we found that substantial antimonate and antimonite were adsorbed on the SRB cell surface, indicating that cell surface is a critical reaction site of Sb transformation and precipitation. Sb was adsorbed to the cell surface by C-C and C=O groups, and was further precipitated by forming Sb_2_S_3_ and Sb_2_S_5_ or was coprecipitated with the P-containing group. Partial Sb(V) reduction was also observed on the SRB cell surface. These results provided a deep insight into the Sb bio-transformation and were an advancement with respect to understanding bioremediation of Sb-contaminated wastewater.

## 1. Introduction

In addition to being used in a wide variety of industries, such as electronics and metallurgy, antimony (Sb) is also known to cause water contamination due to improper handling [1]. Antimony-induced water pollution can be both natural and anthropogenic. The former sources include the weathering of rocks and minerals containing antimony, while the latter sources involve industrial activities, mining, and the discharge of wastewater containing antimony compounds. The non-ferrous metal industry, in particular, accounts for a significant portion in generating a substantial amount of heavy metal-containing wastewater, including antimony (Sb). Some studies have found that nearly 100 years of mining activities have caused severe soil contamination and water pollution in mining areas and these contaminations may alter the stability and functioning of ecosystems [2,3]. The unmitigated release of antimony mine drainage and wastewater has resulted in a persistent escalation of antimony pollutants [4]. It is known to all that the presence of antimony in water poses significant risks to both humans and ecosystems. Studies have shown that antimony (Sb) is listed as one of the major pollutants by the US EPA and the EU EPA due to its potential and proven carcinogenicity, immunotoxicity, genotoxicity, and reproductive toxicity like arsenic, and its toxicity Sb(metal) > Sb(III) > Sb(V), and the soluble compounds of antimony are more toxic than the insoluble compounds [5,6]. When the solubility of antimony in water reaches 3.5 mg/L, it becomes toxic to algae, and when it reaches 12 mg/L, it becomes toxic to fish and shrimps. Long-term ingestion of antimony-contaminated water may cause gastrointestinal disorders, skin irritation, respiratory problems, and may even lead to carcinogenic effects in humans. Antimony and its compounds have been consistently identified as priority contaminants due to their toxicity and biohazardous nature [1,4]. Antimony exhibits toxicity not only to higher organisms such as humans, algae, fish, and shrimps but also to microorganisms. The toxicity of Sb to bacteria, including sulfate-reducing bacteria (SRB), can significantly affect their metabolic activity and heavy metal-precipitation efficiency. Studies have shown that microbial toxicity assays, such as dehydrogenase activity tests and other redox reaction-based methods, provide valuable insights into the impact of toxic substances on microbial communities [7,8]). These methods could help elucidate the potential inhibitory effects of Sb on *Desulfovibrio* species and optimize bioremediation strategies.

The chemical speciation of antimony in water is highly dependent on the surrounding pH. Sb(III) undergoes significant changes in its chemical forms across a pH range of 1 to 12. At pH 1 to 4, the content of compounds in the form of positively charged Sb(OH)_2_^+^ decreases, while the content of compounds in the form of electrically neutral Sb(OH)_3_ increases. Between pH 5 and 9, compounds mainly exist in the form of electrically neutral Sb(OH)_3_. At pH 10 to 12, the content of electrically neutral Sb(OH)_3_ decreases, and compounds in the form of negatively charged Sb(OH)_4_^−^ become more prevalent [9].

In order to mitigate the negative impacts of antimony pollution in water, it is essential to seek effective treatment methods. Numerous approaches have been explored in the field of antimony-removal technology, including physical processes (e.g., precipitation, filtration), chemical methods (e.g., flocculation, precipitation), and advanced techniques utilizing adsorption with materials like activated carbon or other specialized materials. Additionally, the use of sulfate-reducing bacteria (SRB) for mediating metal sulfide precipitation is seen as a promising approach for antimony removal [10,11]. The SRB bacteria put into application contain many species such as *Metallobacterium* [12], *Bacillus thiophilus*, and *Bacillus citriodora*. Extensive research has been carried out on the use of sulfate-reducing bacteria (SRB) for heavy metal remediation, and significant achievements have been made [13,14]. However, there are relatively few studies using SRB to treat Sb-containing wastewater. Wang demonstrated for the first time that SRB can convert SO_4_^2−^ to S^2−^ in mine drainage while converting Sb(V) to Sb(III), and verified that the ratio of Sb(V)/SO_4_^2−^ was an important parameter affecting the efficiency of antimony removal [11]. As previously reported, the addition of Fe^2+^ to the SRB system significantly increased the metabolic activity of SRB [15]. Complementarily, the addition of iron scrap and iron oxidizing bacteria (IOB) to the SRB system resulted in 99.98% Sb(V) removal for the Fe + IOB + SRB system [16]. Differences in carbon sources affected the efficiency of antimony removal in desulfurization processes, with the SRB system utilizing ethanol demonstrating a higher removal efficiency of 97.8% [17]. Temperature was also identified as a primary factor controlling microbial Sb reduction [17]. Characterization of the SRB antimony-removal system’s precipitate indicated that Sb(V) was reduced to Sb_2_S_3_ [18,19]. Proteomic analysis showed that the extracellular protein functional groups of SRB were capable of adsorbing and immobilizing Sb(III), with a significant increase in extracellular proteins involved in electron transfer [20]. Despite many studies on Sb precipitation from wastewater by SRB, the effects of SRB on Sb(III)/Sb(V) precipitation in various habitats under anoxic conditions remain unclear, as do the magnitudes of the impacts of different environmental elements on precipitation efficiency. Furthermore, the precipitation mechanism of SRB on Sb(III)/Sb(V) under anoxic conditions requires further elucidation.

In this work, we analyzed the effect of different conditions (temperature, pH, C/N ratio, and SO_4_^2−^ concentration) on the precipitation of Sb(III)/Sb(V) by SRB, and used scanning electron microscopy–energy dispersive X-ray (SEM-EDS), and X-ray photoelectron spectroscopy (XPS) to study the morphology, components, and surface chemistry of SRB before and after the immobilization of Sb(III)/Sb(V) by adsorption and the precipitation mechanism of Sb(III)/Sb(V) from water under oxygen conditions. The aim of this work is to reveal the mechanism related to the fixation of Sb(III) and Sb(V) by SRB. The work provided theoretical and technological foundations for the application of SRB in the remediation of antimony-polluted wastewater.

## 2. Materials and Methods

### 2.1. The SRB Strains and Culture Condition

The strain used in this study, *Desulfovibrio desulfuricans* subsp. CSU_dl, was isolated from underground water and preserved by the Key Laboratory of Biometallurgy of the Ministry of Education at Central South University, China. This sulfate-reducing bacterium is well known for its ability to reduce sulfate to sulfide and plays a crucial role in heavy metal precipitation. The culture conditions of the strain were as follows: anaerobic incubation for 3–5 days, incubation temperature of 30 °C. The lyophilised strain was activated, dissolved in 0.1–0.2 mL of culture medium or sterile water, and inoculated on 1–2 blood agar plates and incubated in an anaerobic incubator. After the colonies grew on the plates, the culture was enriched with modified Barr sulfate liquid medium. The strain was cultured in an anaerobic incubator. All those incubations and water treatment in this study were well controlled in anaerobic conditions by loading 97% N_2_ and 3% H_2_ to exclude oxygen.

### 2.2. Experiment Design

The initial concentrations of Sb(III) and Sb(V) were set at 15, 30, 45, and 60 mg/L. The logarithmic-phase SRB bacterial solution was inoculated into the medium containing Sb(III) and Sb(V) with a 10% inoculum volume. To optimize the precipitation conditions for Sb(III) and Sb(V), five experimental variables were investigated, with each condition performed in triplicate. Anaerobic flasks were incubated on a shaker at 180 rpm for 24 h. After the reaction, SRB cells were collected by centrifugation at 3500 rpm for 20 min. The experimental conditions included the following:

- Temperature: Reaction temperatures were set at 20 °C, 25 °C, 30 °C, and 35 °C.
- Initial pH: The pH was adjusted to 4, 5, 6, 7, and 8.
- Carbon-to-Nitrogen (C/N) Ratio: C/N ratios were set at 20:1, 40:3, 10:1, 8:1, and 20:3.
- Initial Sulfate Concentration: SO_4_^2^⁻ concentrations were set at 800, 1200, 1600, and 2000 mg/L.

### 2.3. Antimony Precipitation Efficiency

The concentration of SO_4_^2−^ in the wastewater during treatment was determined using the barium chromate spectrophotometric method. The procedure was concise and well-organized, as follows: First, 1 mL of a 2.5 mol/L hydrochloric acid solution was added to both the water sample and the standard solution, and the mixture was boiled for approximately 5 min. Next, 25 mL of barium chromate suspension was added to each sample, and the mixture was boiled again for about 5 min. Afterward, the conical flask was removed, and the solution was allowed to cool slightly. Then, ammonia was added drop by drop until the solution turned lemon yellow, followed by the addition of two more drops. The solution underwent filtration using a slow qualitative filter paper, and the resulting filtrate was collected in a 50 mL cuvette. The conical flask and filter paper were rinsed three times with deionized water, and the filtered liquid was collected in a cuvette and was measured at 420 nm. Antimony concentration in wastewater was determined by ICP-AES.

Then, the best conditions for treating the Sb wastewater with SRB were determined. The subsequent experiment was set up as follows: 300 mL solution with 30 mg/L Sb(III) and 45 mg/L Sb(V), pH 7.1 ± 0.1, temperature 30 °C. The Sb-containing wastewater was treated with SRB (inoculation rate 10%) for 4 days. The bacterial cells, water, and precipitates were collected for further analysis.

### 2.4. Microbial Morphology

To detect the microbial morphology, the samples after 24 h treatment were collected by centrifuging. Scanning electron microscopy (SEM) was employed to examine the morphology of the sulphate-reducing bacteria. The specimens underwent double fixation with glutaraldehyde and osmium tetroxide (H_2_[OsO_4_(OH)_2_]), followed by a gradient ethanol dehydration process lasting 15–25 min.

### 2.5. Morphological Structure and Chemical Composition of the Products

After washing and freeze-drying, two sample sets were obtained: untreated SRB organisms and SRB organisms adsorbed with Sb(III)/Sb(V). Scanning electron microscopy–energy dispersive X-ray (SEM-EDX) was used to analyze the morphology and composition of both untreated SRB bacteriophage samples and those with Sb(III)/Sb(V) adsorption. In addition, X-ray photoelectron spectroscopy (XPS) was employed to comprehensively characterize the elemental composition, including C, N, O, P, S, and Sb spectra, for further investigation of the chemical properties on the SRB surface.

## 3. Results and Discussion

### 3.1. Morphology and Growth Characteristics of SRB

**Figure A1:**
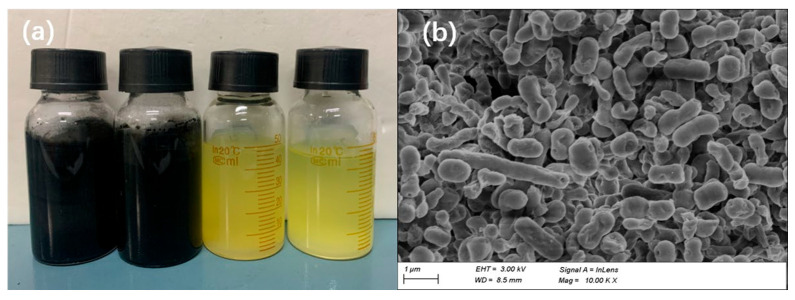
Sulfate-reducing bacteria solution (**a**) and scanning electron microscope image (**b**).

The bacterial liquid was black in color, accompanied by hydrogen sulphide gas. Scanning electron microscope (SEM) observation showed that the SRB bacteria were in the shape of an arc (Figure A1). During the SRB growth, the pH value fluctuated between 7.2 and 7.5, the Eh redox potential was maintained between −55 mV and −70 mV, and the number of bacteria reached the maximum on the 4th day, and then began to decrease on the 5th day due to the accumulation of the product H_2_S. After 7–9 days of incubation, the precipitation effect of SRB on SO_4_^2−^ in the medium was higher than 91%, and a large desulphurization rate was achieved. It was shown that the sulphate-reduction rate of SRB reached a maximum in the pH range of 7.0–7.5, which justified the better desulphurization efficiency of the SRB bacteria used in this experiment [21,22].

### 3.2. Sb(III)/Sb(V) Precipitation by SRB Under Different Conditions

The pH, temperature, carbon to nitrogen ratio, and SO_4_^2−^ concentration exert different impacts on the precipitation of Sb(III) and Sb(V) (Table 1).

***pH*:** For Sb(III), sulphate-reducing bacteria started to precipitate Sb(III) at partial neutrality (pH 6–8). The precipitating efficiencies of Sb(III) at pH 6, 7, and 8 on the seventh day were 98.09%, 91.57%, and 89.89%, while it was 25.89% at the weakly acidic condition (pH 4). Similar findings were observed for Sb(V). At pH 4, Sb(V) precipitated at a rate of 62.06%; at pH 7, it reached a maximum fixation rate of 94.89%; at pH 8, it decreased slightly to 91.21%. The pH values of 6 and 7, respectively, were optimal for the precipitation of Sb(III) and Sb(V) by SRB. The result is similar to a previous study [23], which showed that SRB were less active under acidic conditions and had a higher solution redox potential during growth, which reduced the efficiency of desulphurization and antimony precipitation.

**Figure 1:**
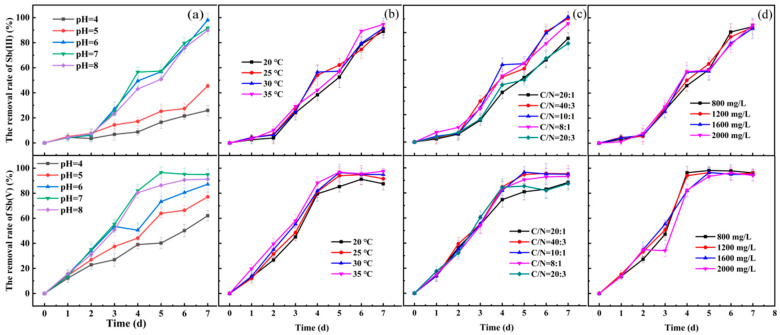
Effects of various parameters on the removal efficiency of Sb(III) and Sb(V) by sulfate-reducing bacteria (*Desulfovibrio desulfuricans*): (**a**) Initial pH (4, 5, 6, 7, and 8) at initial Sb concentration 30 mg/L, temperature 25 °C, C/N ratio 10:1, and sulfate concentration 1600 mg/L. (**b**) Temperature (20, 25, 30, and 35 °C) at initial Sb concentration 30 mg/L, pH 7, C/N ratio 10:1, and sulfate concentration 1600 mg/L. (**c**) Initial C/N ratio (20:1, 40:3, 10:1, 8:1, and 20:3) at initial Sb concentration 30 mg/L, temperature 25 °C, pH 7, and sulfate concentration 1600 mg/L. (**d**) Initial sulfate concentration (800, 1200, 1600, and 2000 mg/L) at initial Sb concentration 30 mg/L, temperature 25 °C, pH 7, and C/N ratio 10:1.

The inhibitory effects of low pH on the desulphurization and antimony precipitation by SRB are varying and complicated. The effect of pH on SRB can be explained in two ways. Firstly, pH has a direct impact on SRB metabolism, disrupting its cellular homeostasis, destroying the pH gradient from the inside and outside of the cell, and ultimately causing energy loss. More protons diffuse through the cell membrane at lower pH levels than at neutral pH due to diffusion pressure across the membrane. To cope with low pH stress, SRB employ both active and passive mechanisms to maintain pH homeostasis. Active mechanisms include proton pumps, which actively expel excess H⁺ ions from the cell using ATP, and amino acid decarboxylases, which produce neutralizing amines through decarboxylation reactions. Passive mechanisms involve changes in membrane lipid composition to reduce proton permeability and the expression of positively charged surface proteins that buffer external H⁺ ions. These strategies help stabilize intracellular pH but divert energy from growth and metabolism, thereby impacting SRB performance in acidic environments. More energy from redox processes was needed to maintain pH homeostasis if the pH gradient in the extracellular and intracellular environments was too great [24]. Second, the pH changed the forms of some elements found in the environment, including organic acids, heavy metals, and sulfides. The forms of sulphur metabolites (H_2_S, HS^−^ and S^2−^) present in the solution of sulphate-reducing bacteria are dependent on the pH of the solution, and in acidic solutions (pH 4.5–5.27), the main form of sulfide substances present was molecular H_2_S (~99%), which tended to volatilize easily [25], and therefore desulphurization for Sb(III) precipitation at a pH of 4.57 was less efficient. Under the condition of a neutral environment, the aqueous solution had a high concentration of sulfide, which increased the probability of Sb(III) binding with sulfide [26]. The SRB metabolism was stronger, so the neutral environment was more conducive to the desulfurization and antimony precipitation from wastewater by SRB. As shown in Figure 1a, as the initial pH increased, so did the removal of SO4^2−^ and Sb(V). This might be the result of a higher pH, a drop in H^+^ concentration, less competition, and a larger amount of heavy metal ion adsorption on the active sites, which would increase the removal of heavy metal ions [27].

***Temperature***: Figure 1b shows the effect of different temperature conditions on antimony precipitation. On the 7th day, the precipitation efficiency of SRB for both Sb(III) and Sb(V) exceeded 90% at 25–35 °C. The precipitation efficiency of SRB for Sb(III) was 91.56%, 88.97%, and 94.60%, respectively. The precipitation efficiencies were 91.56%, 88.97%, and 94.60% for Sb(III) and 91.56%, 94.89%, and 97.53% for Sb(V). The combined findings demonstrated that 35 °C was the ideal temperature for both Sb(III) and Sb(V) precipitation by SRB in this investigation. A previous study concluded that SRB reduced thiosulfate at a growth temperature of 40–50 °C. However, when the growth temperature reached 30°C, virtually no H_2_S was produced [28]. And the results showed that the ideal growth temperature range for SRB was 25 °C to 35 °C, which was consistent with this study.

There are two main ways that temperature affects Sb precipitation by SRB. On the one hand, temperature is an important influencing factor for the growth of SRB. Temperature modifies the microorganism’s own enzyme activity, which in turn affects the efficiency of Sb(III) fixation. Certain enzymes in these cells will be inhibited by too high or too low a temperature, which will negatively impact cell growth and product synthesis, as well as alter cell morphology, metabolic function, and microbial toxicity, or even cause cell death [29]. Additionally, low temperature caused the lipids in the cell membrane to wax and the activity of membrane proteins to decrease, which limited the cell membrane’s ability to transport electron donors and electron acceptors. This was the primary mechanism by which low temperature affected SRB metabolism [30]. On the other hand, the solubility of H_2_S in wastewater was influenced by temperature; high temperatures make H_2_S less soluble in wastewater, which lessens H_2_S’s inhibitory effect on SRB [31].

***C/N ratio*:** The C/N ratio represents the relative availability of carbon and nitrogen sources in the medium, which is a critical factor affecting bacterial growth and metabolism. Different C/N ratios in the substrate have certain effects on microbial growth. Reasonable adjustment of the C/N ratio in the substrate was one of the efficient measures to accelerate microbial growth and promote microbial action. The C/N ratio of Sb(III) and Sb(V) were shown in Figure 1c. Both high (20:1) and low (20:3) C/N ratio affected the growth of SRBs and their efficiency of desulphurization and antimony precipitation, and the immobilization rates of Sb(III) were 90.26%, 91.57%, and 86.66% for C/N ratios of 40:3, 10:1, and 8:1, respectively. When the C/N ratio was 40:3, the Sb(V) precipitation rate reached the highest value of 95.28%, which was the optimal C/N ratio.

At a low C/N ratio (e.g., 20:3), Sb(V)-removal efficiency decreased significantly due to limitations in microbial growth and metabolism. Microbial stoichiometry and metabolic theory state that when a substrate’s low C/N ratio (or high N availability) satisfies microbial requirements or when the compounds are easily broken down by the microorganisms, increasing the production of enzymes and the breakdown of organic carbon, microbial activity is higher [32]. Therefore, the selection of an appropriate carbon-to-nitrogen ratio in microbial growth media can alleviate the metabolic constraints of microorganisms in heavy metal environments and enhance the efficiency of heavy metal fixation. Research has demonstrated that the growth and metabolism of SRB were restricted by a low C/N ratio [33]. In contrast, a high C/N ratio, insufficient nitrogen, low buffering capacity of the digestive solution, and easy decrease in pH caused the effectiveness of SRB’s removal of Sb to decline. The selection of an appropriate carbon-to-nitrogen ratio is critical to alleviating metabolic constraints in microorganisms under heavy metal stress. While a low C/N ratio can inhibit Sb(V) removal by limiting SRB activity, excessively high C/N ratios can lead to nitrogen insufficiency, low buffering capacity, and pH reductions, all of which also reduce Sb(V) removal efficiency.

***SO_4_^2−^ concentration*:** Increase in SO_4_^2−^ concentration had little impact on the Sb(V) and Sb(III) immobilization efficiency of SRB; moreover, the Sb(V) immobilization efficiencies were higher than 94% in the range of sulphate concentration of 800–2000 mg/L. The results indicated that the immobilization efficiency of Sb(V) in the range of 800–2000 mg/L was higher than 94%. Meanwhile, the precipitation rate of Sb(III) under each SO_4_^2−^ concentration condition was above 91.02%.

The SO_4_^2−^ concentration in the solution directly reflects the equilibrium relationship between the substrate and microorganisms, and is also the main indicator of the system’s ability to reduce sulfate ions. The production of hydrogen sulfide through heterogeneous sulfate reduction, or sulfide generation, is the fundamental metabolic characteristic of SRB. Sulfate and sulfite can be the electron acceptors for SRB during heterogeneous sulfate reduction [34]. When sulfate levels are sufficient, sulfate serves as the main electron acceptor for SRB metabolism; in situations where sulfate levels are insufficient, sulfite serves as the primary electron acceptor. It was demonstrated that SRBs can grow normally despite very low sulfate concentrations, and that microorganisms in the environment can propel this low-sulfate lake’s sulfur recovery to high levels [35].

In this study, the SRB strain could grow well under the Sb(III) concentration of 30 mg/L. Under anaerobic conditions, when the Sb(III) concentration is 30 mg/L, the temperature is 30 °C, the pH value is 7.13, the C/N ratio is 10:1, and the SO_4_^2−^ concentration is 1600 mg/L; the removal rate of Sb(III) by SRB could reach up to 91.02%. For Sb(V), the SRB strain could grow well under 60 mg/L of Sb(V). A lower toxicity of Sb(V) compared to Sb(III) would lead to the tolerance concentration of Sb(V) being higher than that of Sb(III). Under anaerobic conditions, when the Sb(V) concentration is 45 mg/L, the temperature is 35 °C, the pH value is 7.24, the C/N ratio is 40:3, the SO_4_^2−^ concentration is 2000 mg/L, and the removal rate of Sb(V) by the SRB reaches 94%. Even though the precipitation rate was a bit lower than some previous studies which reached up to 98.7% removal rate [10], the initial Sb concentration was much higher in our study than other studies (45 mg/L vs. 20 mg/L). Much more Sb was removed in our study than previous studies. The results indicate that the *Desulfovibrio desulfuricans* CSU_dl strain had excellent Sb tolerance, as well as bioremediation potentials.

### 3.3. Characterization of Precipitates Generated by SRB

**Figure 2:**
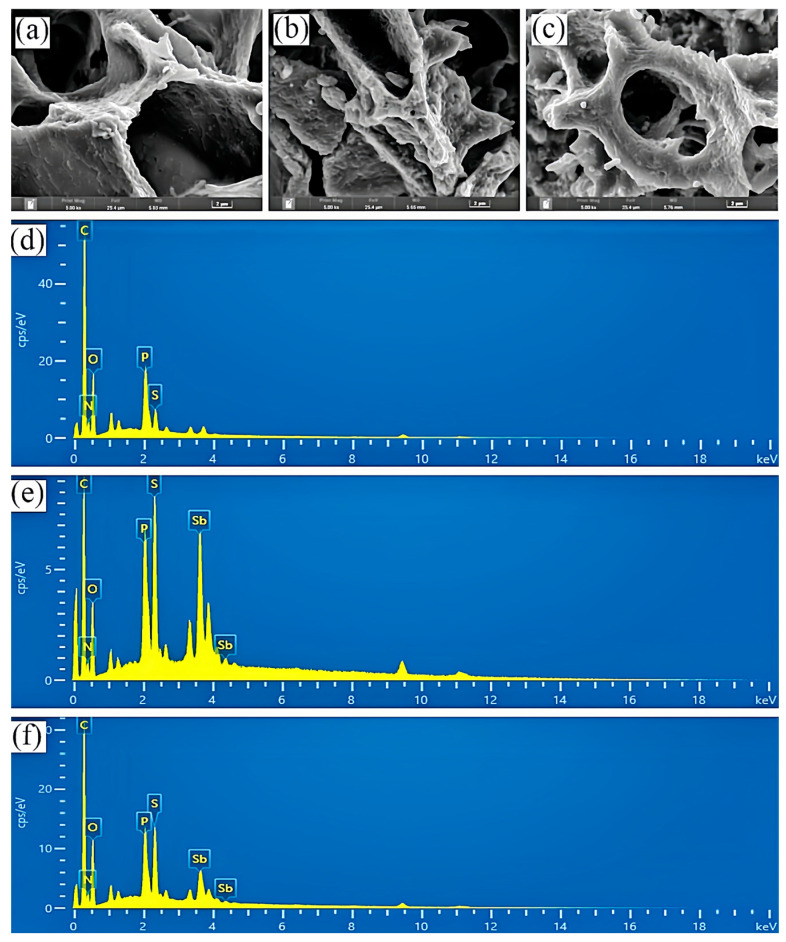
SEM images of freeze-dried SRB bacteria solution (**a**), SRB-Sb(III) immobilized products (**b**), SRB-Sb(V) immobilized products (**c**); EDM images of freeze-dried SRB bacteria solution (**e**), SRB-Sb(III) immobilized products (**d**), SRB-Sb(V) immobilized products (**f**).

The morphology and chemical composition of Sb(III)/Sb(V) precipitates on the SRB cell surface were investigated using SEM-EDS (Figure 2). The SRB cells exhibited a smooth surface with significant pore cavities and thin pore walls. Chemical composition analysis of the SRB cell surface indicated that C, P, O, S, and N were the primary elements. After immobilization, Sb(III) adsorption on the SRB cell surface resulted in morphological changes, including a rougher surface, blocked pore cavities, thicker pore walls, and the formation of ellipsoidal insoluble material. The corresponding surface chemical analysis revealed that C, S, P, Sb, O, and N were the main elements in the reaction products. These observations suggest that Sb(III) precipitation occurred on the SRB cell surface, producing antimony trisulphide (Sb_2_S_3_) as the primary product. The mechanism driving Sb(III) precipitation is hypothesized to involve mineralization mediated by cell wall surface materials, as represented by the following reaction (Equation (1)):

3S^2−^ + 2Sb(OH)_3_ + 6H^+^ → Sb_2_S_3_(s) + 6H_2_O (1)

The mechanism of Sb(V) precipitation by SRB organisms (Equations (1) and (2)) is inferred from the above results, where part of Sb(V) were reduced to Sb(III), and mineralization occurred under the action of cell wall surface substances to produce antimony trisulphide and antimony pentasulphide, resulting in the precipitation of Sb(V) from the wastewater by SRB.

5S^2−^ + 2Sb(OH)_6_^−^ + 12H^+^ → Sb_2_S_5_(s) + 12H_2_O (2)

Cell morphology analysis by SEM indicated that the precipitation of Sb occurred mainly on the cell surface. In particular, abundant P elements were observed on the cell surface as indicated by EDS analysis. This could be due to P being the essential elements for the cell membrane. P could co-precipitate with heavy metals, which further promoted the precipitation of Sb. The results were further supported by the XPS and FTIR analysis.

### 3.4. Analysis of Surface Functional Groups Involved in the Adsorption and Immobilization of Sb(III)/Sb(V) by SRB

**Figure 3:**
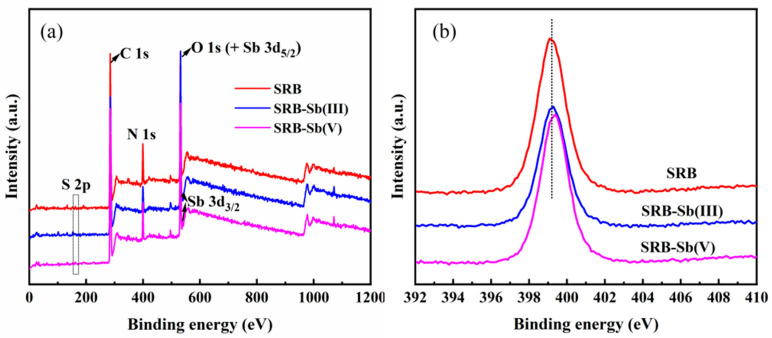
The XPS survey spectra of different SRB before and after Sb(III)/Sb(V) adsorption: (**a**) full spectrum; (**b**) N 1s orbital part spectrum. The red line represents the XPS analysis of the surface of normal SRB cells, the pink line represents the XPS analysis of SRB cell surfaces after Sb(V) adsorption, and the blue line represents the XPS analysis of SRB cell surfaces after Sb(III) adsorption.

The chemical composition, elemental valence, and binding forms of SRB before and after immobilization of Sb(III)/Sb(V) by adsorption were investigated by XPS analysis. As shown in Figure 3, the SRB adsorption-immobilized Sb(III)/Sb(V) products clearly identified the peaks of P 2p, S 2p, C 1s, N 1s, O 1s, and Sb 3d_5/2_ in the scanning spectra, with the peaks of Sb 3d_5/2_ and O 1s being very close to each other at the binding energy of 528 eV–530.3 eV. The characteristic peak of Sb 3d_3/2_ was also identified in the product of SRB adsorption of immobilized Sb(III). The S 2p peak corresponds to a binding energy of 164.3 eV, and its increase indicated the production of sulphides in the product. This suggests that sulphate-reducing bacteria react with Sb(III) or Sb(V) in solution, allowing antimony to be adsorbed onto the product surface, which remains consistent with our previous predictions.

On the basis of the shape of the XPS absorption peak in the N 1s orbital fractionation spectrum (Figure 3b), the binding energy of the N 1s peak is 399.08 eV in the SRB treatment, 399.10 eV in the SRB-Sb(III) treatment, and 399.38 eV in the SRB-Sb(V) treatment, suggesting that the binding energy of the N 1s peak in SRB to Sb(V) biosorption immobilization is shifted to the right, indicating a possible loss of electrons. This may be due to the metal ion chelating with the N atom in the amino and imine groups and the pair of lone electrons in the N atom being shared with the metal ion, resulting in a lower electron cloud density and a higher binding energy peak for the N atom [36].

**Figure 4:**
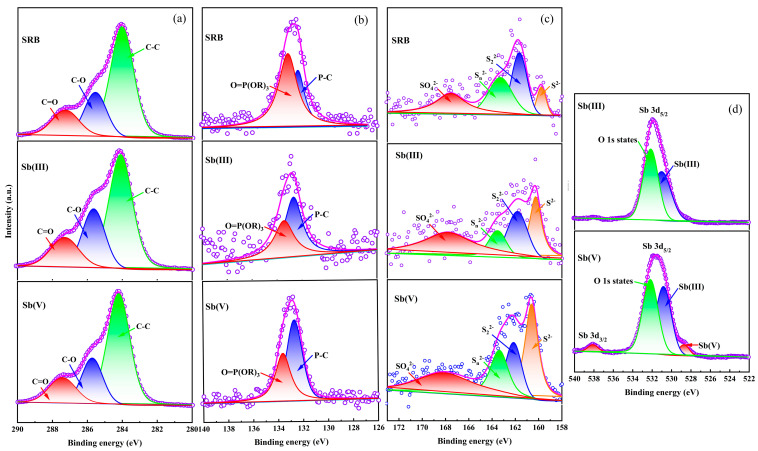
The XPS spectrum of different SRB before and after Sb(III)/Sb(V) adsorption (**a**) C 1s, (**b**) P 2p, (**c**) S 2p, (**d**) Sb. The pink line represents the total XPS spectra of each element, while the different filled lines represent the XPS spectra of the various chemical bonds of that element.

Three peaks originally made up the C 1s orbital fractionation of the XPS absorption peaks (Figure 4a), which corresponded to C-C (284.08 eV), C-O (285.58 eV), and C=O (287.28 eV). The amount of C-O increases and the amount of C-C and C=O decreases following the adsorption immobilization of Sb(III) and Sb(V), as can be observed, suggesting the participation of carbonaceous groups in the Sb adsorption immobilization process.

The P 2p orbital spectra of the XPS absorption peaks (Figure 4b) initially consisted of two peaks corresponding to O=P(OR)_3_ (133.18 eV) and P-C (132.28 eV), respectively. It can be seen that after the adsorption and immobilization of Sb(III)/Sb(V), the O=P(OR)_3_ content decreased and the P-C content increased, and the P-C content exceeded the O=P(OR)_3_ content, indicating that there is a relevant chemical reaction occurring between Sb(III)/Sb(V) and phosphorus-containing groups, and that phosphorus-containing functional groups play an important role in the adsorption and immobilization of Sb, which is consistent with the results of previous research [37].

The S 2p channel splitting spectrum of the XPS absorption peaks (Figure 4c) initially consisted of four peaks corresponding to S^2−^ (160.68 eV), S_2_^2−^ (162.38 eV), S_n_^2−^ (163.98 eV), and SO_4_^2−^ (167.88 eV). Significant increases in S^2−^ and decreases in S_2_^2−^ and S_n_^2−^ were seen in Sb(III) and Sb(V); after Sb(III) was immobilized, SO_4_^2−^ increased, and after Sb(V) was immobilized, SO_4_^2−^ dropped. Sb 3d_3/2_, which does not overlap with the O 1s, was employed as a guide for the fitting of the Sb 3d_5/2_ peak because the Sb 3d_5/2_ peak overlaps with the O 1s. Therefore, the spin-orbit splitting and ratio of the Sb 3d_3/2_ peak dictate the strength and binding energy of the Sb 3d_5/2_ peak. The two characteristic peaks in Figure 4d for Sb(III) and Sb(V) at the binding site during Sb(V) adsorption show that Sb(V) was partially decreased during adsorption. The reduction reaction, however, is thought to occur during the adsorption of Sb(V) by SRB, with S_2_^2−^ and S_n_^2−^ in solution and their surface functional groups acting as electron donors, reducing Sb(V) to Sb(III) and immobilizing it by binding to functional groups to form mineralized substances. This is supported by the presence of the Sb(III) peak at 530.88 eV.

## 4. Conclusions

Due to the massive mining and utilization of antimony-mining resources, the surrounding water and soils are seriously polluted. The sulfate-reducing bacteria (SRB) method has the advantages of high efficiency, low cost, and no secondary pollution, and is highly advantageous in the treatment of antimony-contaminated water and soil. In this study, we find that under anoxic conditions, the precipitation rate of Sb(III) wastewater treated by SRB reached more than 91.02% when the Sb(III) concentration was 30 mg/L, temperature was 30 °C, pH was 7.13, C/N ratio was 10:1, and SO_4_^2−^ concentration was 1600 mg/L. When the Sb(V) concentration was 45 mg/L, the temperature was 35 °C, the pH was 7.24, the C/N ratio was 40:3, and the SO_4_^2−^ concentration was 2000 mg/L, the precipitation rate of Sb(V) from the wastewater by SRB was more than 94%. The morphology and precipitation of antimony are largely influenced by abiotic factors that restrict the growth of SRB bacteria. However, these factors do not work exactly the same for Sb(III) and Sb(V). The cell surface is the main activate site for Sb adsorption and precipitation. Sb was adsorbed to the SRB cell surface by the C-C and C=O group; the P-containing group also played a role in adsorption and precipitation of SRB. The adsorbed Sb was then precipitated by forming the sulfide precipitation. In this process, S_2_^2−^ and S_n_^2−^ and their surface functional groups acted as electron donors, reducing a part of Sb(V) to Sb(III) and combining with functional groups. Mineralization occurred on the surface of SRB cells, generating Sb_2_S_3_ and Sb_2_S_5_ solid precipitates, thus achieving the precipitation of Sb(III)/Sb(V) from wastewater by SRB. However, this study used artificially generated wastewater, which may be different to real conditions. A further study using real wastewater with complex pollutants is also needed to promote the SRB remediation technology.

## Figures and Tables

**Table 1:** Precipitation of Sb(III)/Sb(V) by SRB under different conditions on day 7.

		Precipitation Rate of Sb(III) (%)	Precipitation Rate of Sb(V) (%)
pH	4	25.89 ± 4.20	62.07 ± 4.20
	5	45.44 ± 2.64	77.13 ± 3.23
	6	98.09 ± 5.35	87.17 ± 6.10
	7	91.57 ± 5.06	94.89 ± 4.37
	8	89.89 ± 3.45	91.21 ± 3.89
Temperature (°C)	20	88.97 ± 2.65	87.54 ± 5.06
	25	91.53 ± 5.56	91.56 ± 3.55
	30	91.57 ± 8.06	94.89 ± 4.37
	35	94.60 ± 2.65	97.53 ± 2.66
C/N	20:1	75.77 ± 4.20	88.36 ± 5.05
	40:3	90.26 ± 4.64	95.28 ± 6.55
	10:1	91.57 ± 4.06	94.89 ± 4.37
	8:1	86.66 ± 3.35	93.22 ± 6.39
	20:3	72.07 ± 4.45	87.46 ± 5.45
SO_4_^2−^ (mg/L)	800	92.92 ± 2.65	96.27 ± 2.37
	1200	92.42 ± 5.56	95.97 ± 2.36
	1600	91.57 ± 8.06	94.89 ± 4.37
	2000	94.23 ± 4.25	94.16 ± 4.66

**Figure A2:**
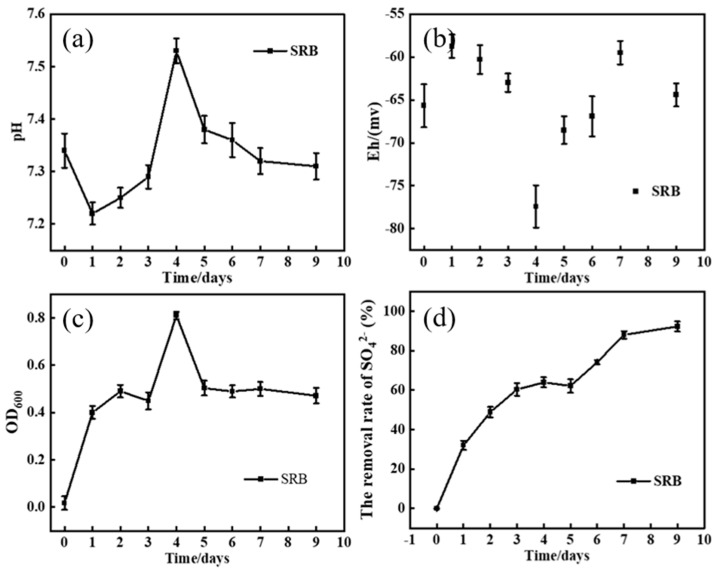
Parameter changes during the growth of sulfate-reducing bacteria (**a**) pH, (**b**) Eh, (**c**) OD_600_, (**d**) SO_4_^2−^ removal efficiency.

## Data Availability

The data supporting the reported results are available from the corresponding author upon reasonable request.
